# Characterization of a Novel Amphiphilic Cationic Chlorin Photosensitizer for Photodynamic Applications

**DOI:** 10.3390/ijms24010345

**Published:** 2022-12-25

**Authors:** Margarita A. Gradova, Oleg V. Gradov, Anton V. Lobanov, Anna V. Bychkova, Elena D. Nikolskaya, Nikita G. Yabbarov, Mariia R. Mollaeva, Anton E. Egorov, Alexey A. Kostyukov, Vladimir A. Kuzmin, Irina S. Khudyaeva, Dmitry V. Belykh

**Affiliations:** 1 N.N. Semenov Federal Research Center for Chemical Physics, Russian Academy of Sciences, 119991 Moscow, Russia; 2 Emanuel Institute of Biochemical Physics of Russian Academy of Sciences, 119334 Moscow, Russia; 3 Institute of Chemistry, Komi Scientific Center, Ural Division of the Russian Academy of Sciences, 167982 Syktyvkar, Russia

**Keywords:** chlorin photosensitizers, photodynamic therapy, solubilization studies, protein binding, singlet oxygen generation, photostability, intracellular distribution, phototoxicity

## Abstract

A novel amphiphilic cationic chlorin *e*_6_ derivative was investigated as a promising photosensitizer for photodynamic therapy. Two cationic –N(CH_3_)_3_^+^ groups on the periphery of the macrocycle provide additional hydrophilization of the molecule and ensure its electrostatic binding to the mitochondrial membranes and bacterial cell walls. The presence of a hydrophobic phytol residue in the same molecule results in its increased affinity towards the phospholipid membranes while decreasing its stability towards aggregation in aqueous media. In organic media, this chlorin *e*_6_ derivative is characterized by a singlet oxygen quantum yield of 55%. Solubilization studies in different polymer- and surfactant-based supramolecular systems revealed the effective stabilization of this compound in a photoactive monomolecular form in micellar nonionic surfactant solutions, including Tween-80 and Cremophor EL. A novel cationic chlorin *e*_6_ derivative also demonstrates effective binding towards serum albumin, which enhances its bioavailability and promotes effective accumulation within the target tissues. Laser confocal scanning microscopy demonstrates the rapid intracellular accumulation and distribution of this compound throughout the cells. Together with low dark toxicity and a rather good photostability, this compound demonstrates significant phototoxicity against HeLa cells causing cellular damage most likely through reactive oxygen species generation. These results demonstrate a high potential of this derivative for application in photodynamic therapy.

## 1. Introduction

Photodynamic therapy (PDT) is a rapidly developing area of treatment of different pathologies including cancer [1,2,3,4], atherosclerosis [5,6,7], and infectious diseases [8,9,10,11]. This method involves the generation of reactive oxygen species (ROS) by a photoexcited photosensitizer (PS) molecule, leading to oxidative cell damage. The main advantages of PDT include low invasiveness and the absence of systemic side effects, while the limitations of this method originate from the limited light penetration through the biological tissues, oxygen deficiency in tumor cells, and incomplete selectivity of the PS accumulation in the target tissues [12]. Thus, the therapeutic efficiency of PDT strongly depends on the type of PS. An ideal PS must combine several properties, such as intense absorption within the “therapeutic window” (600–850 nm), sufficient photostability and bioavailability, high singlet oxygen quantum yield, low dark toxicity, and rapid clearance from the body [13]. Despite the numerous potential PSs synthesized in recent decades, the selection of the most efficient PS still remains a challenge.

Cationic chlorins, along with porphyrins and phthalocyanines, are promising PSs for the PDT of various neoplasms [14,15,16] and the photoinactivation of pathogenic microorganisms [17,18]. Their low dark toxicity, intense absorption in the near-IR spectral range, and high singlet oxygen quantum yields provide good therapeutic efficacy of PDT, whereas the presence of cationic functional groups promotes binding to the mitochondrial membranes and cell walls of Gram-negative microorganisms. To date, there is an active search for compounds combining the advantages of both natural and synthetic PSs. Examples of such compounds are cationic chlorin *e*_6_ (Ce6) derivatives containing a phytol fragment at the macrocycle periphery [19], which increases their affinity towards membranous structures. At the same time, a clear spatial separation of the hydrophilic and lipophilic parts of the molecule ensures its amphiphilicity and the ability to self-assemble or integrate into various supramolecular assemblies. The latter may include nanocarriers capable of targeted drug delivery which significantly improve the efficiency of PDT due to the PS solubilization and selective accumulation in proliferating tissues [20,21,22,23,24,25,26].

In this work, we studied a novel dicationic Ce6 derivative (Compound **1**) with two quaternized aminomethyl groups and one phytol fragment which significantly enhances the tendency of aggregation in polar aqueous media. In this regard, it was necessary to find supramolecular systems capable of stabilizing this compound in polar media in a photoactive monomolecular form. For this purpose, micellar solutions of nonionic surfactants, polymer solutions (including serum albumin), as well as polymeric micelles and polyelectrolyte complexes were used. We also evaluated the intracellular distribution and in vitro cytotoxic activity of this compound.

## 2. Results and Discussion

### 2.1. Solubilization Studies

**Figure 1:**
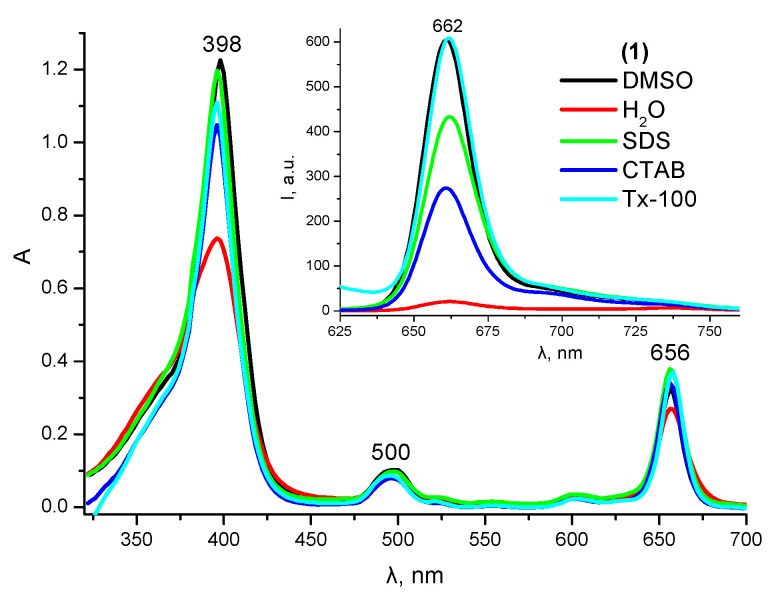
UV–Vis absorption and fluorescence (insert) spectra of **1** in DMSO, H_2_O, and micellar surfactant solutions.

In a pure organic solvent dimethyl sulfoxide (DMSO), Compound **1** is characterized by the presence of two intense absorption bands: λ_B_ = 396 nm (lgε_B_ = 5.27) and λ_Q_ = 656 nm (lgε_Q_ = 4.81), with the latter band being in the transparency range of biological tissues (Figure 1) and corresponding to an intense fluorescence band with λ_em_ = 662 nm (Figure 1, insert). In an aqueous medium, due to the presence of a non-polar phytol residue in the molecule, Compound **1** exhibits characteristic signs of aggregation (decreased molar absorption coefficients, increased half-width of the absorption bands, and strong fluorescence quenching). However, in the presence of model surfactants with a concentration above the critical micelle concentration (cmc), the partial or complete restoration of the spectral and luminescent parameters of the monomolecular form of **1** is observed (Figure 1).

Among the different surfactants tested, the most effective solubilization of **1** is observed in the micelles of a nonionic surfactant (Triton X-100). Similar results were obtained during solubilization studies with other nonionic surfactants, such as Brij-58, Tween-80, and Cremophor EL (Figure S1). The last two solubilizers are biocompatible, and hence can be used to create a stable dosage form of 1. For this reason, further studies of the photostability and photochemical activity of 1 in aqueous media were carried out in micellar solutions of the above nonionic surfactants.

For a qualitative assessment of the possible localization area of **1** within the nonionic surfactant micelles, we used a technique described in [27,28] involving the study of the luminescence quenching efficiency of the solubilized chlorin with I^−^ ions. In this case, since the access of the iodide anions is limited by the near-surface hydrated layer of the micelles, the fluorescence intensity changes of chlorin during the titration of the micellar solution with potassium iodide can provide evidence for the fluorophore localization area in the micelles. The data obtained by this method indicate the peripheral localization of **1** at the interface between the hydrophobic core and the polyoxyethylene shell in nonionic surfactant micelles (Figure S2). These results correspond to the conclusions obtained earlier by the other authors on chlorins of a similar structure [27,28].

### 2.2. Photostability and Photochemical Activity

**Figure 2:**
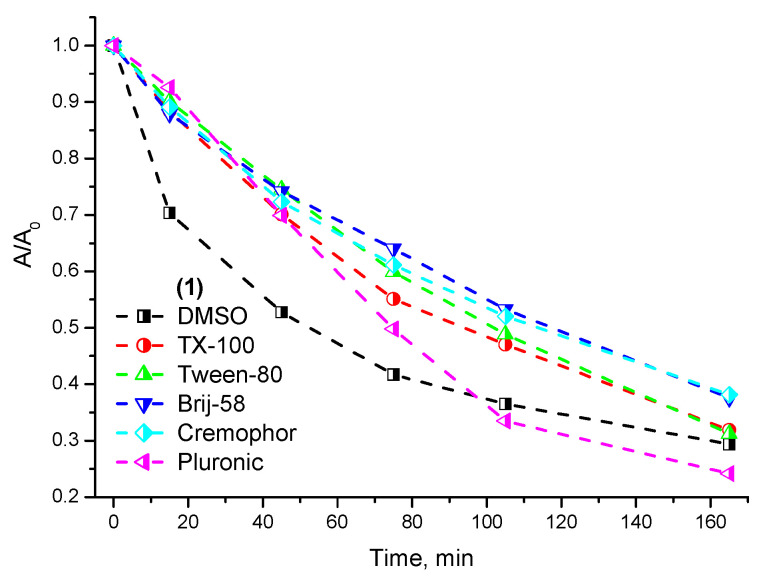
Photostability of **1** in micellar nonionic surfactant solutions.

For a successful application of the compound as a photosensitizer, it is necessary to ensure its high photostability. A comparison of the resistance to photobleaching for Compound **1** solubilized in the micelles of various nonionic surfactants indicates its effective stabilization in TX-100, Tween-80, Brij-58, and Cremophor EL micelles (τ_1/2_ = 100 min) compared with its solution in DMSO (τ_1/2_ = 60 min) (Figure 2). At longitudinal irradiation periods (more than 60 min), a high photodegradation rate of **1** is also observed in the micelles of an amphiphilic polymer Pluronic F-127 (τ_1/2_ = 80 min), which is in good agreement with the previously obtained data on its low solubilization efficiency towards **1** (Figure S1).

In addition to the high photostability of a PS, its singlet oxygen quantum yield is of crucial importance for the efficiency of PDT since ^1^O_2_ is the major type of ROS causing photoinduced cell death through the oxidative damage of the membrane lipids and nucleic acids. Singlet oxygen generation proceeds through a type II photodynamic mechanism involving a direct energy transfer from the triplet excited state of the PS to the molecular oxygen, which is also an essential component in photodynamic therapy. An accurate estimation of Φ_Δ_ can be performed either through a direct method by intrinsic ^1^O_2_ IR phosphorescence measurements at 1270 nm [29] or by an indirect “chemical trapping” method involving different singlet oxygen quenchers [30,31]. In this study, we used the latter method with 1,3-diphenylisobenzofuran (DPBF) as a selective ^1^O_2_ acceptor. According to the above method, Φ_Δ_ for **1** in DMSO was found to be 0.55 ± 0.05.

**Figure 3:**
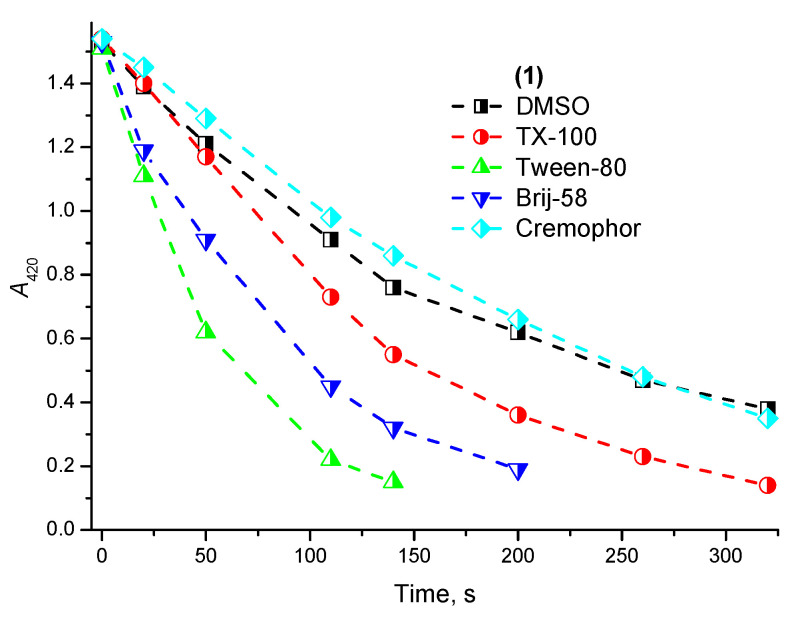
Singlet oxygen generation efficiency for **1** in DMSO and micellar nonionic surfactant solutions.

Unfortunately, an accurate determination of the absolute value of Φ_Δ_ for the PSs in micellar media by the above method is impossible due to the complex system of equilibria existing in aqueous microheterogeneous systems and different localization areas of the PS and the chemical trap molecules. However, it is possible to qualitatively compare the rate of DPBF photosensitized oxidation with singlet oxygen in the presence of **1** in micellar systems. Such a comparison indicates the key importance of the surfactant type for the photochemical activity manifestation of the solubilized PS in vitro. The highest photochemical activity for **1** was observed in Tween-80 micelles, which confirms the advantages of such nanocarriers for the solubilization of various chlorin derivatives observed earlier [32,33]. The lowest ^1^O_2_ generation efficiency was observed in Cremophor EL (Figure 3). This may be due to the greater number of the double bonds in Cremophor EL molecules undergoing oxidation by the singlet oxygen produced, hence resulting in the underestimation of ^1^O_2_ generation efficiency.

### 2.3. Triplet State Investigation

**Figure 4:**
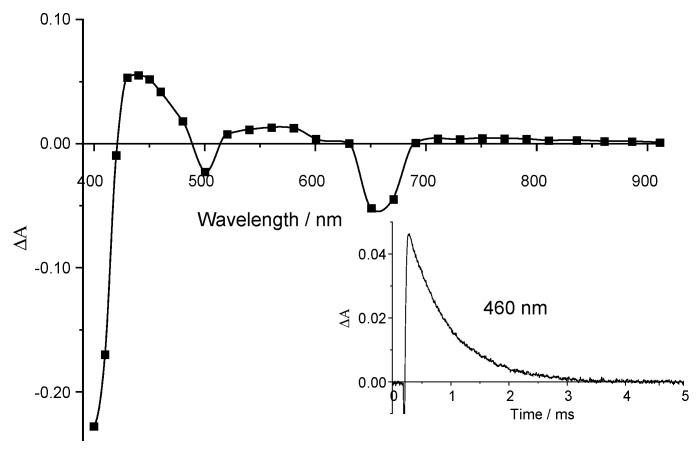
Triplet–triplet absorption spectrum of Compound **1** (5 × 10^−7^ M) in DMSO (240 μs after flash). Insert: Compound **1** triplet decay kinetics at 460 nm.

Conventional flash photolysis experiments were conducted to obtain triplet state spectral and kinetic characteristics. Upon the photoexcitation of Compound **1** degassed solution in DMSO, the intensive triplet absorption band at the 430–480 nm region appears while the bleaching of the Soret (400–420 nm) and Q-bands (500 nm, 650–670 nm) is observed (Figure 4). The triplet state kinetics (Figure 4, insert) are monoexponential with the rate constant *k*_T_ = 1.4 × 10^3^ s^−1^.

Laser flash photolysis experiments were conducted to determine the quenching constant (*k*_q_) of the Compound **1** triplet state by molecular oxygen that is calculated according to the equation:

*k* = *k*_T_ + *k*_q_[O_2_] (1)

where *k* is the observed triplet state decay rate constant in air-equilibrated solution (s^−1^), *k*_T_ is the triplet state decay rate constant in the absence of oxygen (s^−1^), *k*_q_ is the bimolecular quenching constant of the triplet state by oxygen (M^−1^s^−1^). As *k*_T_ << *k*, we can take *k*_T_ ≈ 0, then *k* = *k*_q_[O_2_]. Taking into account that oxygen solubility in DMSO is 2.1 mM [34] and *k* = 6.0 × 10^5^ s^−1^, the quenching constant estimated is 2.9 × 10 ^8^ M^−1^s^−1^. The diffusional rate constant for DMSO (*k*_diff_ = 3.3 × 10 ^9^ M^−1^s^−1^) was calculated using [35]. Considering the spin-statistical factor of 1/9 (3.7 × 10 ^8^ M^−1^s^−1^) the obtained *k*_q_ value is close to 1/9*k*_diff_, which indicates the efficient triplet state quenching process.

The triplet quantum yield for Compound **1** was calculated by a comparative method using the laser flash photolysis technique [36]. As a reference, standard ZnTPP was employed (Φ_T_ = 0.83 and *ε*_T_ = 7.4 × 10 ^4^ M^−1^ cm^−1^) [36]. A triplet state extinction coefficient at 440 nm for Compound **1** was determined using the singlet depletion method considering singlet state extinction at 396 nm to be ε_S_ = 1.86 × 10 ^5^ M^−1^ cm^−1^. This resulted in ε_T_ = 4.5 × 10 ^4^ M^−1^ cm^−1^. For both compounds, optical densities at the excitation wavelength were matched, and a linear dependence between the laser intensity and maximum triplet absorption was established. The obtained Φ_T_ value is 0.58 ± 0.1. The singlet oxygen quantum yield determined for Compound **1** in DMSO (Φ_Δ_ = 0.55) corresponds with the calculated Φ_T_ value and indicates an efficient triplet state oxygen quenching process by the novel photosensitizer **1**.

### 2.4. Interaction with Polymers

**Figure 5:**
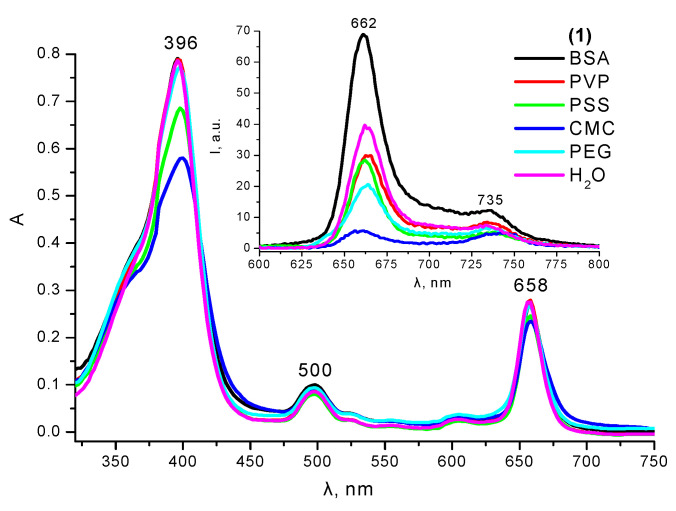
UV–Vis absorption and fluorescence (insert) spectra of **1** in polymer aqueous solutions.

In addition to solubilization in the surfactant micelles, binding with various water-soluble polymers was also studied for **1**. It has been shown that the ionic binding of cationic chlorin **1** with polyanions – poly(sodium-*p*-styrenesulfonate) (PSS) and carboxymethyl cellulose (CMC) leads to significant fluorescence quenching due to the increasing probability of the nonradiative deactivation of the PS singlet excited state due to the interchromophore interaction in the system of the neighboring macrocycles bound to the close sites of the polymer chain (Figure 5). In this case, the maximum binding efficiency is observed near the equimolar ratio of the chlorin **1** molecules and anionic groups of the polymer.

Among other water-soluble polymers, the interaction of **1** with neutral poly-*N*-vinylpyrrolidone (PVP) and polyethylene glycol (PEG) was observed, which did not significantly affect the absorption spectra but also led to fluorescence quenching (Figure 5). The appearance of an additional emission band at 735 nm, according to the titration experiments (Figure S3), results from the aggregate formation. In this regard, such polymers cannot be considered as effective stabilizers of the photoactive monomolecular form of **1** in aqueous media, despite their high stabilization efficiency with respect to a number of other more hydrophilic Ce6 derivatives [37,38]. The most noticeable changes in the fluorescence spectra were observed upon the binding of **1** to bovine serum albumin (BSA), the main transport protein in the blood plasma. The protein binding of the PSs usually enhances their bioavailability, increases circulation time in the bloodstream, and preserves photoactivity in biological media.

### 2.5. Protein Binding

**Figure 6:**
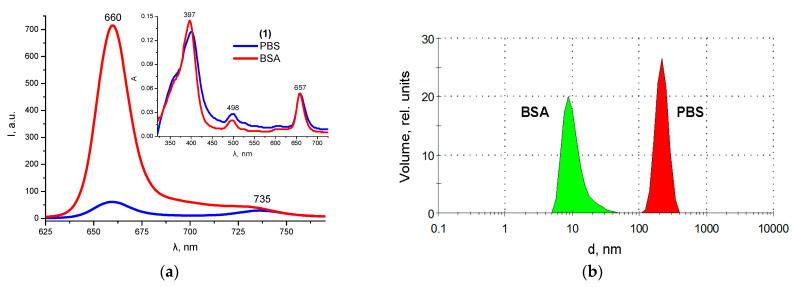
Comparison of fluorescence and absorption (insert) spectra (**a**) and particle size distribution (**b**) for **1** in pure PBS and in the presence of BSA.

The complexation of **1** with albumin slightly influences the band shape and the maxima position in the absorption spectrum of chlorin but is accompanied by a significant fluorescence intensity increase in the latter (Figure 6a). The formation of chlorin-albumin complexes is also confirmed by the dynamic light scattering data. The average hydrodynamic particle diameter in albumin solutions in the presence of **1** is only 10 nm, while in the absence of protein in a phosphate-buffered solution, chlorin **1** predominantly exists in the aggregated state with an average aggregate particle size of about 250 nm (Figure 6b).

**Figure 7:**
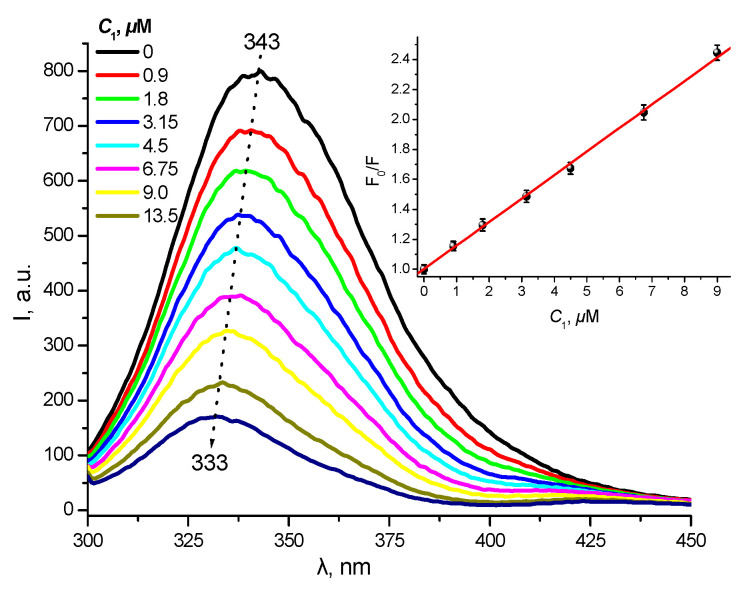
BSA fluorescence quenching upon titration with **1** (λ_ex_ = 290 nm, *C*_BSA_ = 5 μM) and the corresponding Stern–Volmer plot (insert).

A more detailed quantitative study of the complex formation in the chlorin–albumin system allowed to establish the binding parameters, in particular, to estimate the binding constant *K*_b_ = 3.65 × 10^5^ M^−1^, as well as to calculate the Stern–Volmer constant *K*_SV_ = 1.55 × 10^5^ M^−1^ for the protein fluorescence quenching in the presence of **1** (Figure 7). The linear Stern–Volmer dependence shown in Figure 7 (insert) indicates one type of binding in the chlorin–BSA system. A more detailed study of the albumin binding sites responsible for the complex formation with **1** will be further performed using the competitive titration method. However, one can suppose the crucial role of the hydrophobic phytol residue in the amphiphilic chlorin binding to the albumin molecule.

According to the titrations performed, BSA solutions with protein concentrations above 0.5 mg/mL (which is 5–10% of its physiological concentration in blood) provide effective stabilization of the monomolecular fluorescent form of **1** with the stoichiometric molar ratio Chlorin:BSA varying between 1:3 and 2:3 (Figure S4). The results obtained reveal the possibility to use the natural transport systems for binding **1** and delivering it to the target tissues. However, further experiments on the photosensitizer binding to the different blood plasma components are required.

### 2.6. Polyelectrolyte Complexes

**Figure 8:**
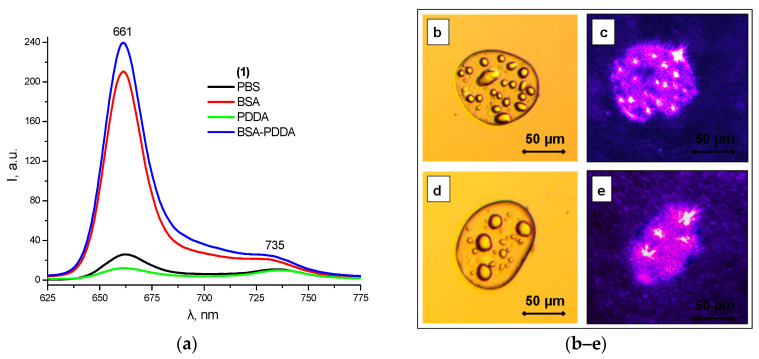
Fluorescence spectra of **1** in PBS, BSA, PDDA, and BSA-PDDA PEC (**a**) and optical micrographs of coacervate microdroplets of BSA-PDDA PEC (**b**–**e**).

The above-described data on the protein binding of **1** made it possible to synthesize novel photoactive polyelectrolyte complexes (PECs) between BSA, which is a polyanion at physiological pH (pI 4.9), and a synthetic polycation poly(diallyldimethylammonium chloride (PDDA), with the PS **1** strongly bound to albumin. Such PECs with the molar ratio of BSA:PDDA 3:1, containing chlorin **1** in a monomolecular form, demonstrate intense fluorescence according to the optical microscopy data (Figure 8a). The average diameter of the chlorin-containing microdroplets is 50 microns (Figure 8b). The resulting phase-separated photoactive supramolecular systems based on self-assembled polyelectrolyte complexes containing a photosensitizer can be considered as models of primitive photosynthetic protocells [39,40].

### 2.7. In Vitro Biological Tests

**Figure 9:**
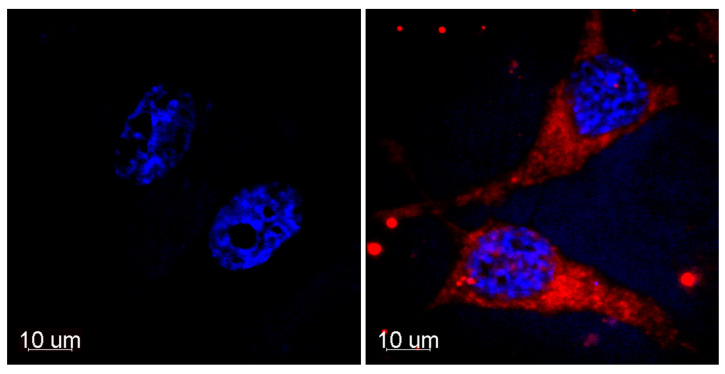
Intracellular distribution of **1** (red) in HeLa cells after incubation for 2 h (right). Untreated control (left). Blue–nuclei stained with Hoechst 33342. The sections were created at the middle height of the cells.

In the framework of the preliminary biological tests in HeLa cancer cells, dark and light cytotoxicity, as well as intracellular distribution, were evaluated for Compound **1**. We observed an intense red fluorescence of the cells incubated with **1** at 665 nm. Red fluorescence was mainly registered in the cytoplasm and in perinuclear areas after incubation for 2 h (Figure 9). The predominant cytoplasmic distribution may evidence the partial colocalization of **1** with mitochondria, which is consistent with the previous reports describing Ce6 specificity to these organoids, promoting the mitochondrial damage [41,42].

**Figure 10:**
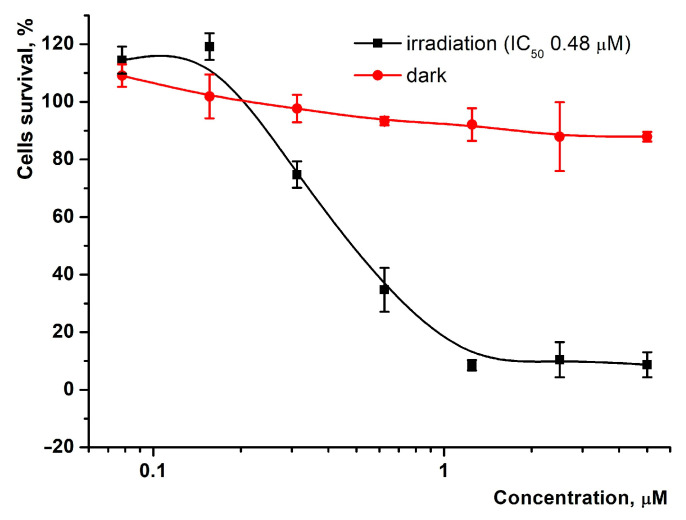
Light-induced and dark cytotoxicity of **1** analyzed by MTT assay. The percentage of cell viability was determined relative to the viable control cells. Survival of HeLa cells was measured at 72 h after treatment.

We also evaluated the light-induced and dark cytotoxicity of **1** against HeLa cancer cells by applying the MTT test (Figure 10) [43,44].

Preliminary cytotoxicity studies of **1** allowed to choose optimal PS concentrations, irradiation mode, and solubilization conditions, which did not influence the cell culture. As shown in Figure 10, up to 90% of the cells remained viable after treatment with **1** in the absence of light, indicating low dark toxicity. The irradiation stimulated photodynamic activity resulting in a strong cytotoxic effect (IC_50_ = 0.48 µM) against HeLa cells, suggesting that this compound can be considered as a promising anti-tumor photosensitizer. However, the above results should be confirmed by further in vivo studies.

## 3. Materials and Methods

### 3.1. Synthesis of Compound **1**

**Figure 11:**
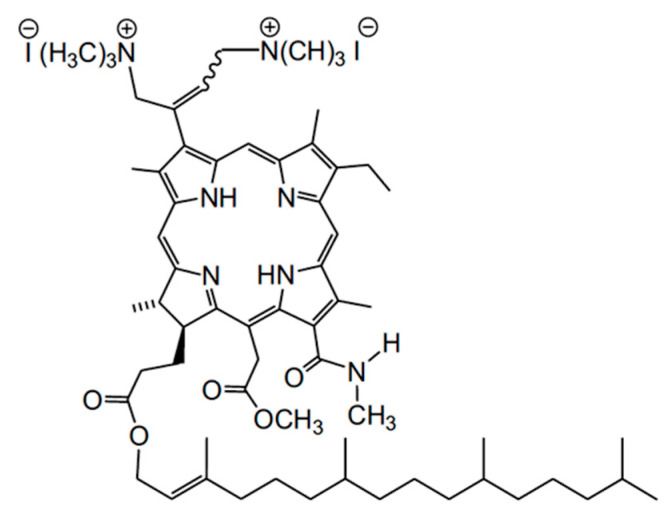
Structure of Compound **1**.

3(1),3(2)-bis(*N*,*N*,*N*-trimethylaminomethyliodide) chlorin *e*_6_ 13(1)-*N*-methylamide-15(2)-methyl,17(3)-phytyl ester (Compound **1**, Figure 11) was synthesized from pheophytin *a* extracted from the air-dry spirulina according to the procedure described earlier [19].

Briefly, two aminomethyl groups were introduced by the aminomethylation of the vinyl group of a 13-methylamide derivative by bis(*N*,*N*-dimethylamino)methane, similarly to the previously described procedure for the synthesis of phytol-free cationic Ce6 derivatives [45,46]. As in the latter case, aminomethylation resulted in a mixture of cis- and trans-isomers forming in a 1:1 ratio. The formation of cationic groups was carried out by the alkylation of the nitrogen atoms of the dimethylaminomethyl groups. The structure of the compound obtained was confirmed by UV–Vis-, IR-, and ^1^H NMR spectroscopy. According to the NMR data, a phytol fragment does not change during all the chemical modifications performed.

^1^H and ^13^C NMR spectra were recorded on a Bruker Avance II 300 spectrometer (Bruker, Germany) (300.17 and 75.5 MHz, respectively) in CDCl_3_ at room temperature. Diffuse reflectance IR spectra were recorded on a Shimadzu IR Prestige 21 FT-IR spectrometer (Germany) in KBr pellets. Electronic absorption spectra were recorded on a Shimadzu UV-1700 instrument (Japan) in 10 mm quartz cells in CHCl_3_. ESI mass spectra were recorded on a Thermo Finnigan LCQ Fleet instrument (USA). The reaction process was monitored by TLC on Sorbfil plates (IMID, Krasnodar, Russia). Silica gel with a particle size of 0.06–0.2 mm (Alfa Aesar, Haverhill, MA, USA) was used for column chromatography. Tetrahydrofuran and bis(*N*,*N*-dimethylamino)methane were preliminarily distilled, while the other solvents and reagents were used without any further purification.

### 3.2. Solubilization Studies

Polymer and surfactant solutions were prepared from the commercially available reagent grade chemicals: sodium *n*-dodecyl sulfate (SDS, Scharlau, Spain), cetyltrimethylammonium bromide (CTAB, BioChemica & AppliChem, Germany), Triton^®®^ X-100 (TX-100, Merck, Germany), Brij^®®^-58 (Sigma Aldrich, St. Louis, MI, USA), Tween-80 (Sigma Aldrich, USA), Cremophor^®®^ EL (Sigma Aldrich, USA), Pluronic^®®^ F-127 (Sigma Aldrich, USA), polyethylene glycol (PEG, M.W. 1000, Merck, Germany), poly-*N*-vinylpyrrolidone (PVP, M.W. 10000, Sigma Aldrich, USA), poly(sodium-*p*-styrenesulfonate) (PSS, M.W. 70,000, Sigma Aldrich, Shanghai, China), carboxymethyl cellulose (CMC, M.W. 250,000, DS = 0.9, Acros Organics, France), poly(diallyldimethylammonium chloride) (PDDA, M.W. < 100,000, Sigma Aldrich, USA), and bovine serum albumin (BSA, Sigma Aldrich, USA) using bidistilled water. Albumin was diluted with phosphate-buffered solution (PBS) with pH 7.4 and ionic strength of 10 мM. Micellar solutions were prepared with a concentration five times higher than the critical micelle concentration (cmc). Solubilization studies were performed according to the following procedure: 10 μL of the stock chlorin solution in DMSO was added under stirring to 3 mL of the solubilizer. The final system with *C*_1_ ~ 5 μM was studied after equilibration for about 30 min.

### 3.3. Instrumental Characterization

#### 3.3.1. UV–Vis Spectroscopy

Electronic absorption spectra were recorded on a HACH DR-4000V instrument (USA) in the wavelength range of 320–800 nm with a step of 1 nm. Fluorescence spectra were recorded on a Perkin Elmer LS-50 luminescent spectrometer (USA) in 10 mm quartz cells with a step of 0.5 nm at room temperature. The excitation wavelength corresponded to 500 nm, and the absorbance in this band did not exceed 0.1. All the measurements were performed at 298 K.

#### 3.3.2. Flash Photolysis

The transient triplet–triplet absorption spectrum was measured using a conventional flash photolysis setup (optical path length 20 cm, excitation was performed through the red optical absorption filters with transmission >620 nm, 80 J/15 µs). Signals were recorded by a PMT-38 photomultiplier (MELZ, USSR) at 400–700 nm. For the NIR region, a similar setup was used with an OPT-101 BURR-BROWN photodiode. The degassing of the solution was provided with the vacuum pump.

#### 3.3.3. Laser Flash Photolysis

Laser pulse photolysis was carried out on an LKS 80 (Applied Photophysics, Leatherhead, UK) laser pulse photolysis setup. The third harmonics of an Nd-YAG laser (Brilliant B, Quantel) were used for excitation. The excitation wavelength in the range of 410–600 nm was tuned by an OPO (MagicPrism, OPOTEK Inc., Carlsbad, CA, USA). The kinetics of the transient species generated by a 5 ns laser excitation pulse was registered by difference absorbance changes in the spectral range of 400–750 nm using a 150 Xe arc lamp with a 50-fold beam overdrive by a 1.5 ms capacitor discharge. The detection system was equipped with a 600 MHz oscilloscope (Agilent Infiniium 10 GS/s) and an R928-type PMT. The kinetic data were processed by the global analysis by fitting the kinetic traces over the whole range of the registration wavelengths with the multiexponential equation:

(2) $$\Delta A_{\lambda }=\sum \Delta A_{\lambda i}\exp(-t/\tau_{i}),$$

where ∆*A*_λ_ is the overall difference absorbance at the registration wavelength λ, ∆*A*_λi_ is the absorbance of the *i*-th transient species at the registration wavelength λ, and *τ*_i_ = 1/*k*_i_ is the lifetime of the *i*-th transient species, with *τ*_i_ and ∆*A*_λi_ being the fitting parameters. The accuracy in the lifetime determination was 15%. During the triplet state quantum yield determination, both the reference standard and Compound **1** solutions OD were matched at the excitation wavelength (415 nm). The accuracy of the triplet quantum yield determination was 20%.

#### 3.3.4. DLS Measurements

Particle size distribution was estimated by the dynamic light scattering (DLS) technique using a Zetasizer Nano-S instrument (Malvern Instruments, Malvern, UK) with a helium–neon laser light source (*λ* = 632.8 nm) at a light scattering angle of 173° and a temperature of 25 °C. All the data obtained are the average of at least three independent measurements.

#### 3.3.5. Optical Microscopy

Optical micrographs in transmission and fluorescence modes were obtained using a BS-702B binocular optical microscope equipped with a UCMOS08000KPB USB camera based on a 1/2.5″ CMOS sensor with a resolution of 3264 × 2448 pixels with Altami Studio software. For the registration of the fluorescence excited in the blue channel, the principle of frequency doubling of an infrared GaAlAs source (λ = 808 nm; a Diode Pumped Solid State Laser with an infrared beam) or a GaN laser with a wavelength of 405 nm was used. In the latter case, the problem of the filtration of the primary IR components of the pump source (λ = 808 nm; photon energy ≈ 1.53 eV) was removed.

### 3.4. Photochemical Measurements

#### 3.4.1. Photostability

The stability of Compound **1** (*C*_1_ = 5 μM) towards photobleaching was monitored in quartz cells at room temperature in air-saturated DMSO. The light source was equipped with a 150 W halogen lamp, a three-lens spherical condenser with a reflector and heat and UV filters. The incident light intensity was 10 mW/cm^2^. The absorbance at the Q-band was periodically measured every 30 min during 3 h of the sample irradiation. Photostability was evaluated by the Q-band absorbance decrease.

#### 3.4.2. Singlet Oxygen Generation

Singlet oxygen quantum yield (Φ_Δ_) for **1** in DMSO was measured by the chemical trapping method using 1,3-diphenylisobenzofuran (DPBF) as a selective ^1^O_2_ quencher and zinc(II) phthalocyanine (ZnPc) as a standard (Φ_Δ_ = 0.67 in DMSO) [47,48]. DPBF solution (*C*_DPBF_ = 0.1 mM) was added to the chlorin solution in DMSO in dark immediately before the start of irradiation. In photochemical experiments, a concentration of **1** was maintained at 2 μM. The photochemical experiment was carried out in quartz cells under ambient conditions in air-saturated DMSO solutions using the same irradiation system described above equipped with a cut-off filter with transmittance ≥ 600 nm. The monitoring of the DPBF absorption decrease at 416 nm during the sample irradiation was performed for 5 min. In all the experiments, the sample absorbance in the Q-band region was less than 0.15, and the final DPBF concentration did not exceed 50 μM. The calculation of the singlet oxygen quantum yield (Φ_Δ_) was performed according to the known procedure [47,48] with the error of Φ_Δ_ determination being about 10%.

### 3.5. Biological Tests

#### 3.5.1. Reagents

DMEM culture medium, fetal bovine serum (FBS), and trypsin-EDTA (Gibco, Grand Island, NY, USA); mowiol (Calbiochem, San Diego, CA, USA); culture flasks (25 cm^2^), 96- and 24-well plates from Corning-Costar (USA); 3-(4,5-dimethylthiazol-2-yl)-2,5-diphenyltetrazolium bromide (MTT) and Hoechst 33342 (Sigma-Aldrich, USA).

#### 3.5.2. Cell Culture

Human cervical carcinoma HeLa cell line (ATCC) was maintained in flasks in DMEM medium supplemented with 10% fetal bovine serum and gentamycin (50 µg/mL) in a CO_2_-incubator at 37 °C in a humidified atmosphere containing 5% CO_2_. The cells were replated with trypsin-EDTA solution twice per week.

#### 3.5.3. Intracellular Distribution Analysis

The intracellular localization of **1** in HeLa cells was analyzed by confocal laser scanning microscopy (CLSM). Briefly, 2 × 10^4^ (in 1 mL of DMEM medium) HeLa cells were seeded on the round glass slides in 24-well plates for 24 h. The next day after attachment, the cells were preincubated for 2 h in serum-free media and treated with 120 nM of **1** for another 2 h, rinsed three times with PBS, and stained with 2.2 µM Hoechst 33342. Later, the cells were fixed in 2% paraformaldehyde, rinsed with PBS, and embedded in mowiol. Chlorin and Hoechst 33342 fluorescence intensity were observed with a Carl Zeiss Cell Observer Z1 confocal microscope (Jena, Germany) with 100× magnification (excitation at λ_1_ = 355 nm (Hoechst 33342) and λ_2_ = 635 nm (Compound **1** studied)). The pictures were finally processed for the selection of various color combinations using Photoshop software (Adobe, Mountain View, CA, USA).

#### 3.5.4. Cytotoxicity Analysis

HeLa cells were seeded in 96-well plates (4000 cells per well) 24 h before the experiment and incubated under standard conditions. The derivative was added in triplets in the concentration range of 0.078−5 μM. To determine phototoxicity, the cells were irradiated with a 660 nm light-emitting diode for 20 min with a 25 mW/cm^2^ power LED and then incubated for 72 h. Cell survival was determined using a standard MTT assay [49]: 50 μL MTT in DMEM (1 mg/mL) was added into each well. After cell incubation for 4 h at 37 °C, the medium was removed, and the formazan crystals precipitated were dissolved in 100 μL of DMSO. Following this, the absorption intensity of formazan was measured at 540 nm on a microplate reader. Cell viability was determined as a percent of the untreated control.

## 4. Conclusions

A novel amphiphilic cationic Ce6 derivative was characterized as a possible photosensitizer for PDT. It demonstrated an intense absorption at 656 nm (lgε = 4.81) and fluorescence at 662 nm, significantly high triplet quantum yield (Φ_T_ = 0.58 ± 0.1) and singlet oxygen quantum yield (Φ_Δ_ = 0.55 ± 0.1) in DMSO, and a pronounced tendency towards aggregation in aqueous media. Solubilization studies revealed that the nature of the surfactant molecules strongly influences both the photophysical properties and photodynamic activity of the cationic chlorin in micellar systems with the non-ionic surfactants (Tween-80 and Cremophor EL) being the most effective solubilizing agents. Polyanions, in contrast, induce the aggregation of the cationic chlorin due to electrostatic binding at the neighboring monomeric units of the polymer chain, resulting in the excited state self-quenching, and hence, reduced photochemical activity. This compound demonstrates high photostability and strong binding with the main transport protein–serum albumin which may provide its long circulation time and effective accumulation in the target tissues. Polyelectrolyte complexes were also shown to prevent cationic chlorin from aggregation and enhance its bioavailability. The Ce6 derivative studied demonstrated high phototoxicity against HeLa cells and exhibited mostly cytoplasmic and perinuclear localization, which may evidence the involvement of organoids, especially mitochondria, in the cell death mechanism. This opens new prospects for the application of such cationic amphiphilic chlorin-based photosensitizers both in antimicrobial and antitumor PDT.

## Data Availability

The data presented in this study are available in the electronic supplementary material.

## Supplementary Materials

The following supporting information can be downloaded at: https://www.mdpi.com/article/10.3390/ijms24010345/s1.
