# Supplementary material for: Characterization of a Novel Amphiphilic Cationic Chlorin Photosensitizer for Photodynamic Applications
Source: Int J Mol Sci. 2022 Dec 25;24(1):345. doi: 10.3390/ijms24010345 (PMC9820311; doi:10.3390/ijms24010345)
Supplement: Supplementary file 1 [file ijms-24-00345-s001.zip › ijms-2102437-supplementary.pdf]

Supplementary Information

Characterization of a novel amphiphilic cationic chlorin photosensitizer for photodynamic applications

Margarita A. Gradova, Oleg V. Gradov, Anton V. Lobanov, Anna V. Bychkova, Elena D. Nikolskaya, Nikita G. Yabbarov, Mariia R. Mollaeva, Anton E. Egorov, Alexey A. Kostyukov, Vladimir A. Kuzmin, Irina S. Khudyaeva, Dmitry V. Belykh

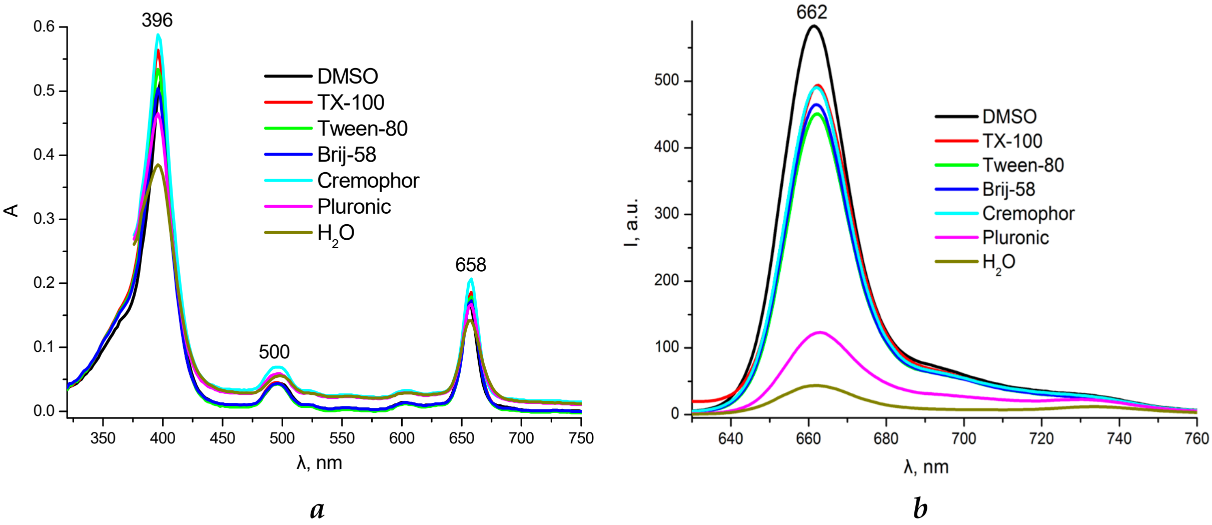

Figure S1. UV-Vis absorption (*a*) and fluorescence (*b*) spectra of **1** in DMSO, H_2O and nonionic surfactant solutions.

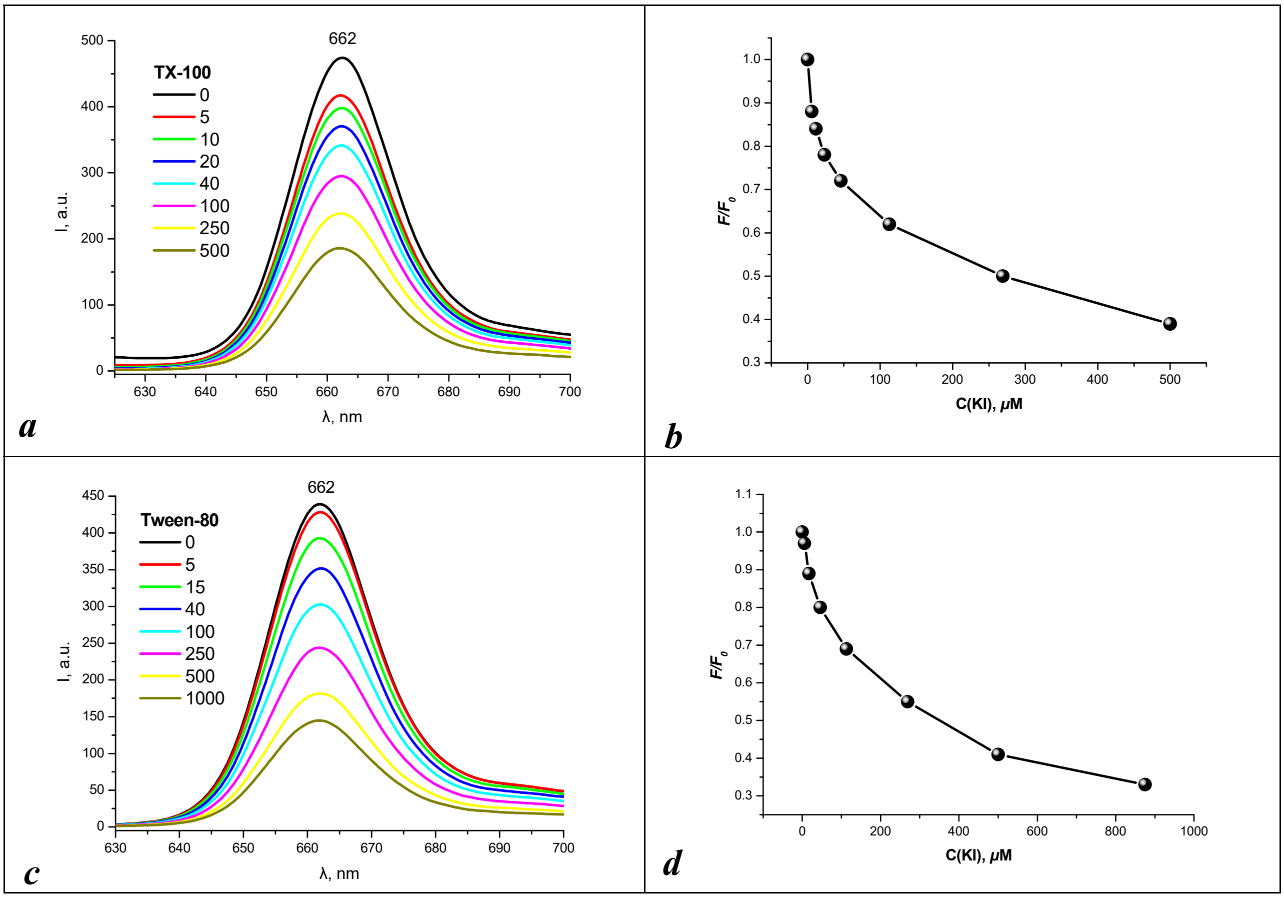

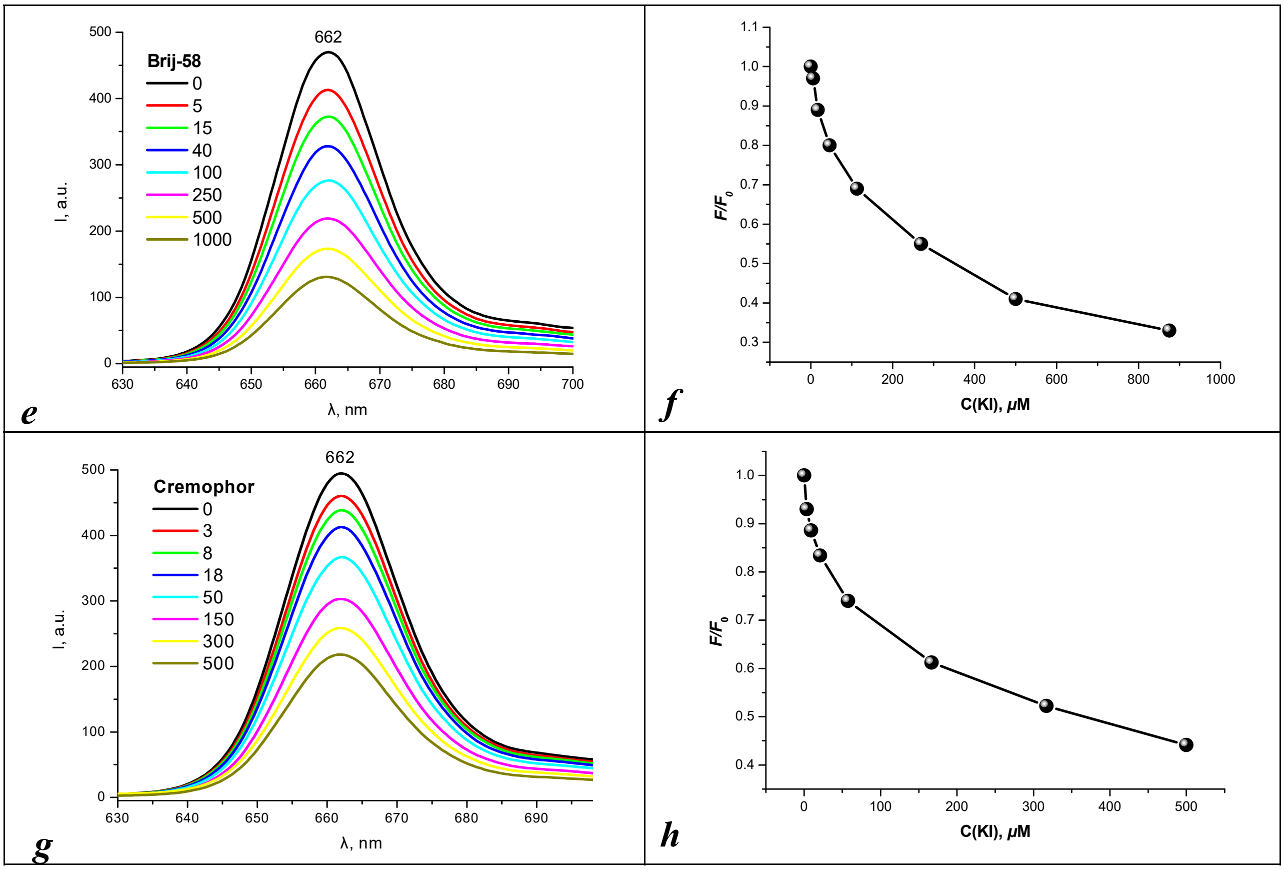

Figure S2. Fluorescence spectra of **1** in nonionic surfactant micelles (***a,c,e,g***) upon titration with KI and the corresponding I^- ion concentration-dependent fluorescence decay curves (***b,d,f,h***).

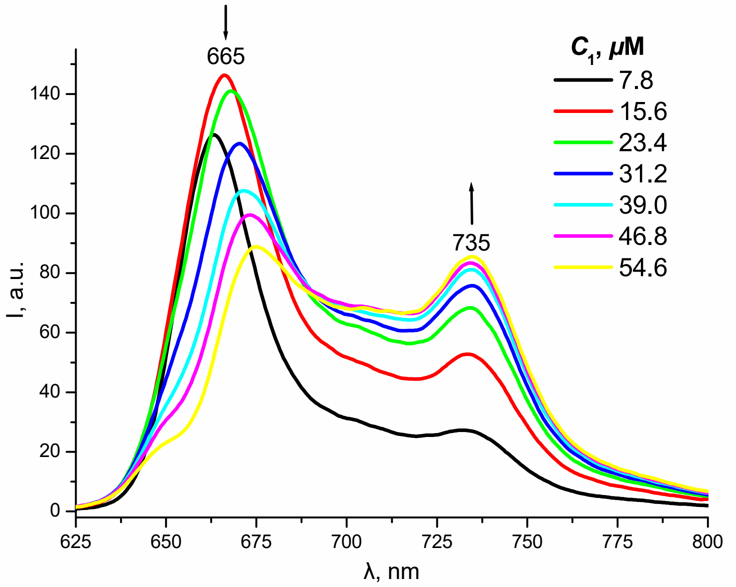

Figure S3. Concentration dependence of the fluorescence spectra of **1** in H_2O with 0.3-2% DMSO.

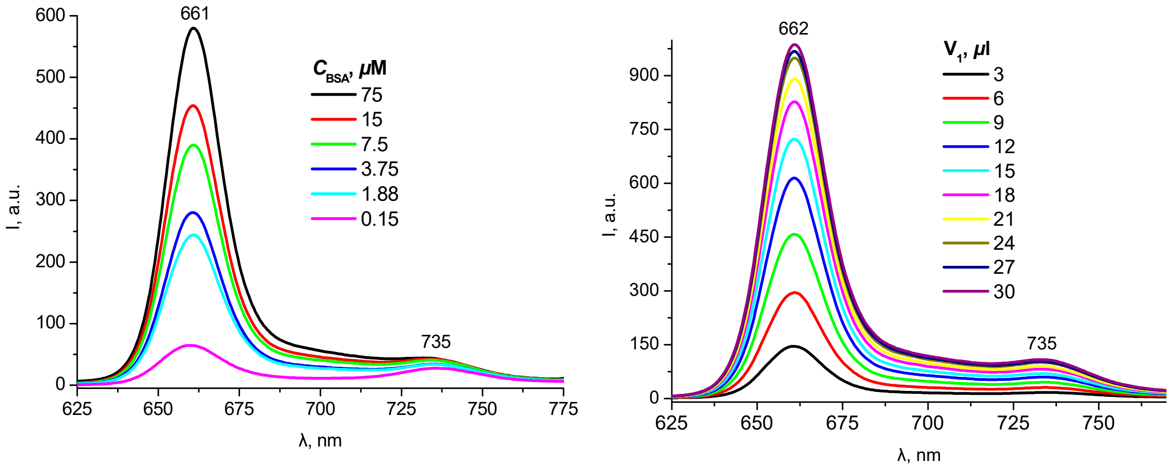

a ***b***

Figure S4. Fluorescence spectra of **1** in BSA at different C_{BSA} (***a***) and different C_1 (***b***).
